# Unmet needs and service satisfaction of victim support for the direct and indirect victims of serious violence: Results from a cross-sectional survey in Taiwan

**DOI:** 10.1371/journal.pone.0192905

**Published:** 2018-02-21

**Authors:** Lanying Huang

**Affiliations:** Graduate School of Criminology, National Taipei University, New Taipei City, Taiwan; DePaul University, UNITED STATES

## Abstract

Victim support services, in mature societies, aim to help victims recover after suffering a traumatic event. The effectiveness of victim support has traditionally been evaluated through rates of service utilization and incidence of psychopathology such as posttraumatic stress disorder. The current study, instead, inquires into service users’ unmet needs and satisfaction, and identifies factors that mediate such subjective measures, using data from a national cross-sectional survey on victims and surviving families of violent crime in Taiwan in 2011. The results reveal: 1) a gap between available and expected services, and 2) a correlation between service utilization and satisfaction, both consistent with previous studies. In addition, the current study identifies unsatisfied service users: They are homicidally bereaved, live with their spouse, suffer from post-crime financial distress and are still waiting for a court verdict on the incident. Victim support that helps victims heal through tailored services incorporating relationship counseling is proposed.

## Introduction

Violent crimes have a tremendous impact upon the victims and their family members, both physically and psychologically. People assaulted by criminal acts are referred to as direct victims, while the family members of the criminally assaulted or killed as “indirect victims” [1].

In many places in the world, including Taiwan, government-funded organizations or “victim support” agencies have been set up to respond to victims’ needs related to therapy, for such post-traumatic psychopathology as delayed grief, complicated grief, and post-traumatic stress disorder [2]. Violently assaulted victims and homicidally bereaved families are usually the target groups and main users of victim support. A survey of more than seven thousand victims of crime in the U.K. indicated that victims of serious offences were more likely to report the need for victim support services than other victims [3]. Another survey in Pennsylvania showed that 64% of victims of violent crime used victim services, compared with only 24% of victims of non-violent crime [4]. The implication is twofold: First, victim service users might be more likely to be victims of serious violence; and second, the evaluation of victim support should differentiate users between violent and non-violent victimization, since the two groups might have different needs. Before a review of previous research findings, an introduction to the current foci of victim support for direct and indirect victims is given, providing the rationale for the victim’s subjective view in the evaluation of victim support.

### Dual aims of victim support: Trauma recovery and Re-victimization prevention

The high needs and usage of victim support by victims of violence have gained much academic attention. One explanation is that a victim’s recovery correlates with their criminal justice experiences, which often increase the need for help and risks of secondary victimization. Compared with victims of other crimes, victims of violence are usually much more exposed to the criminal justice process and less satisfied with the system. Previous studies suggest that both victims and surviving families of homicide might suffer from persistent stress symptoms as a consequence of engaging in the criminal justice process [5, 6]. The criminal justice involvement, which “restricts or delays survivors’ mourning” [7], might be a burden especially for the surviving family of homicide. Therefore, present-day victim support also provides services to ease the sense of secondary victimization during victims’ involvement in the criminal justice system, given the significant empirical evidence confirming the correlation between prolonged trauma reaction and criminal justice experience [8].

The U.S. National Crime Victimization Survey further found that victims who received assistance, compared with those who did not, tended to report more follow-up legal actions [9]. Specifically, the NCVS survey data revealed that more than one fourth of victims who received assistance reported follow-up criminal justice actions, compared with only 4% for the victims who did not receive assistance. In fact, actively seeking justice might be a way of developing a positive coping strategy, if victims have a reliable victim support service on their side. In this sense, helping victims go through the criminal justice system should be and has been among the aims of victim support.

### Evaluation of victim support from users’ perspective

Taiwan enacted the Crime Victim Protection Act in 1998, the first in Asia. Ever since, the Association for Victims Support (AVS, website http://www.avs.org.tw/), a Ministry of Justice-funded victim support agency with 21 regional offices across Taiwan, has been established, as have many public/private non-profit victim support organizations. Eligible groups for the AVS service, initially limited to victims of serious assault and families of homicide, the same group eligible for state compensation claims, were later expanded to include vulnerable victims, such as children victims and human trafficking victims. Nearly two decades have passed since its establishment and it is time to evaluate the efficacy of the victim support services. Previous assessments of victim support often emphasized objective measures such as the rate of victim service utilization and whether services alleviated psychological harm [4, 10–14]. On the other hand, a victim’s subjective attitudes on victim assistance or perceived social support are equally important in enhancing a victim’s esteem, control and sense of safety, which, in turn, reduces subsequent victimization [15], contributing to the improvement of a victim’s wellbeing. Therefore, levels of user satisfaction with victim support are a concurrently interesting topic which deserves more attention than has previously been the case.

The relationship between needs and satisfaction among victim service users appears complex in empirical investigations. Some studies emphasize “satisfaction” by indicating that the majority of victim service users remained satisfied with the services in spite of considering the level of help to be far from sufficient [16]. Others point out dissatisfaction might arise from the discrepancy between needed services and available services. Therefore “meeting needs” should be prioritized [17]. Still others consider many barriers remain that prevent victims from receiving needed services, although more and more services have become available [12, 18].

Using national cross-sectional survey data, this study aims to evaluate victim support for violence victims by including feedback about victim services from targeted service users in Taiwan, namely victims of violent crime themselves and the surviving families of violent crimes. It is hoped that an evidence-based understanding will provide a reference to advance victim service policy and practices. Secondly, to complement the narrow definition in previous studies of victims’ needs as mental health or psychological recovery, this study explores both psychological and practical needs and services.

To the best of this author’s knowledge, no empirical research has ever tried to explore associating factors of unmet needs and dissatisfaction of victim service recipients, nor has any compared service users’ experiences between direct and indirect victims of violent crime. Even less research on victim support in an Asian context can be found. This study intends to fill this theoretical gap by stressing the importance of subjective measures of victim service users. After all, they are those who are most seriously affected by crime.

### Literature review

This study proposes that: (a) the use of service is a continuous spectrum, unlike a dichotomy of use versus non-use, as in most previous studies; and (b) a regression model which includes pre-, peri-, and post-crime independent factors to help explain victim needs and service satisfaction. In what follows, a review of previously identified factors serves to help make assumptions for the regression analysis.

#### Victim service users

Understanding who the victim service users are is of significance in explaining their preferences and attitudes. A survey in Pennsylvania found that the users of victim service programs are characterized as female (65%), high school graduates (51%), not married (57%), and not working full time (42%) [4]. An analysis of historical statistics in the U.S. also found that female victims are more likely to receive assistance than their male counterparts since they are usually more actively seeking and welcoming help [9]. The survey also shows that violent crime victims who have two or more dependent family members under 12 years old are more likely to receive victim services than those who have no dependents or only one. The results of previous empirical studies depict victim service users as vulnerable, disadvantaged, and reactive people who have little informal social support.

#### “Unmet needs” of violence victims/surviving families

The current victim support service model is based on mainly three categories of needs: psychological needs, practical needs, and information needs [19], while information needs have been gradually integrated into victims’ rights legislation. The psychological and practical needs remain key parts, in spite of the fact that the boundary usually blurs. Psychological needs usually include crisis intervention, counseling, therapy, hotline, follow-up support, and trauma recovery. “Practical needs” is an umbrella concept which covers legal, medical, financial, occupational, educational, and security/protection. Previous studies predominantly focused on singular needs, especially mental health service; this lack of diverse needs might hinder our understanding of the utilization and evaluation of victim support services. In particular, practitioners have found that mental health services are seldom the priority for victims in the early days of victimization.

The “mismatch” between victim needs and service provision is a repetitive theme in previous research [16, 20]. Unmet needs were measured via victim’s subjective responses to: (a) would like to have had services/help; (b) would like to have had more or better services/help. Most studies of unmet needs are concerned with victims of violence, rather than victims of non-violent crime, because empirical evidence shows that violently assaulted or seriously wounded victims often express more unmet needs [16]. A review estimated that around 30% to 40% victims of medium to serious offences responded that they would have benefited from the help they failed to obtain readily [21]. Our understanding of needs and unmet needs could also be augmented by a small but growing number of qualitative studies on homicide-surviving families [5, 22, 23]. Focus groups of surviving families and friends conclude that biological, psychological, spiritual and social needs are generally perceived unmet regardless of the amount of time had passed since the incident [23].

#### Evaluating and modeling victim services

In terms of evaluating victim services, researchers usually differentiate the mediating factors into pre-crime, peri-crime, and post-crime variables. Pre-crime factors are demographic characteristics including age, marital status, family members, educational attainment, employment and income. Peri-crime factors include the type of violence, victimization outcome, and victimization impact. Post-crime factors are often the types of informal and formal social support utilization.

Pre-crime factors are often considered control variables in the evaluation model. An analysis of witness satisfaction survey in the U.K. revealed that victim satisfaction was not linked with their age or gender [24]. In some cases, victims’ vulnerability will influence their help-seeking behaviors, eliciting more practical and psychological needs. For example, a study showed that victims who lived with their spouse or partner might be less likely to use victim support since they are able to get assistance from the intermediate relatives [4]. Research on 926 victims also confirmed that single or divorced females used more mental health services [11]. Another survey conducted by Davis and colleagues on 470 victims found employed victims reported more unmet needs since they often demanded more support to participate in the criminal justice process, such as employer notification or child care [16]. In sum, empirical evidence has been mixed, and the relationship between demographic characteristics and victims’ feedback remains an open question.

Victimization experience, including the event and immediate aftermath, is usually crucial in predicting victim needs and satisfaction. An earlier market survey in the U.S. showed that victims of assault had more unmet needs when compared with victims of property crime [16]. In the U.K., victims of serious offences are more likely to report unmet needs than overall victims [3]. A longitudinal survey of victims of violence went further to conclude that these victims reported more use of mental health services and lower satisfaction [25]. It is assumed that suffering from more serious crime and crime impact might generate greater need and/or create dissatisfaction.

Post-crime factors were less mentioned in previous studies, but sometimes changes in victim status in later stages might cause vulnerability, influencing their needs and satisfaction. Survey data showed that crime victims who had moved in the last four years responded more initial needs and unmet needs than respondents with stable residences [16]. Significant life events, such as moving, which are more common among crime victims, suggest that victims cut themselves off their informal social support networks, which might cause social isolation or reduce perceived social support.

## Materials and methods

As there are relatively few studies that investigate the experiences and views of victim support from the perspective of the victims of violence, the current study represents a chance to lessen the scarcity. Specifically, the study explores the characteristics of victim service users, identifies the factors influencing perceived unmet needs and satisfactory level of those services, and, finally, adds to the understanding of victim support in an Asian context. The factors explored in this paper include demographic characteristics, victimization and its impacts, pattern of service utilization, and feedback about services from a survey of 312 direct and indirect victims of violent crime in Taiwan. In order to identify the influencing factors of victims’ perceptions of the services, two regression models were developed.

### Cross-sectional survey of crime victims

Both seriously wounded victims and homicide survivors in Taiwan are automatically referred by prosecution offices to local victim support agencies for compensation claim inquires and other services. In a continuous effort to improve the protection system, a survey project to understand the status of victim support was launched by the Ministry of Justice (Taiwan) in 2011. In the survey, every eligible victim with full contact information in the AVS database was contacted, first by mail and later by telephone, for consent to a face-to-face interview. As a result, 391 (31.5%) victims and 1,617 (27.7%) victims’ families successfully completed the survey in the period between July and November of 2011.

This survey was approved by the intramural project review committee of the Department of Prevention, Rehabilitation and Protection of the Ministry of Justice of Taiwan on March 9, 2011. Collection, process, including de-identification, and the use of the survey data were also overseen by the intramural project review committee, in compliance with the Personal Information Protection Act of Taiwan. The interviewees gave written consent before the survey began. The consent form informed the participants about survey purposes, confidentiality, voluntary principle, rights of consent withdrawing, and the ways of sharing the survey results.

As mentioned, the victims of violence were more likely to be found under-served, and the current research focuses on the victims of non-traffic violence (*N* = 312), namely, the seriously wounded victims (*N* = 73), families of seriously wounded victims who were incapable of answering questions (*N* = 48), and the surviving families of non-traffic homicide (*N* = 191). The first type of victim is direct victims while the rest are indirect victims. This current analysis excluded victims of traffic related injury and death since previous study has pointed out different needs between traffic and non-traffic violence death [26].

### Variables and measurement

#### Dependent variables

Unmet needs and satisfaction are treated as two dependent variables in this paper; the former as the outcome indicator and the latter as the process indicator for the evaluation of services from the users’ perspectives. Unmet needs are quantified as the number of items specified by the victims that they would like to receive but did not receive before the time of interview. In the survey, victims were asked “do you have any unmet needs before, during, or after the judicial process?” and requested to multiply cross 15 items of unmet needs, which were divided into three groups (before, during, and after the judicial process), each in turn with five service items (legal, financial, living, psychological, and medical). In subsequent regression analysis, unmet needs were dichotomously coded, namely with or without unmet needs.

The level of satisfaction was measured in the survey by seven questions pertaining to the perceived quality of services, namely timely service information, sufficient information, transparent procedure, multiple channels, speedy provision, professional staff, and empathetic staff. In each of the above questions, the answers were measured by a Likert-type scale from mostly disagree, disagree, neutral, agree, to mostly agree. High values of Cronbach’s alpha were found for the set of satisfaction questions (α = 0.974 from the victims and α = 0.957 from the families), suggesting reliability of the questions. The level of satisfaction was calculated from sum of the Likert scales and treated as a numerical value in the following linear regression analysis.

#### Independent variables

The predictive variables fall into four domains: demographic factors, incident-related factors, victimization impact factors, and service usage factors. Demographic variables include victim’s gender, education, employment, cohabitants, and dependents. It is hypothesized that neither unmet needs nor satisfactory levels are associated with the demographic characteristics of the victims/family members.

Incident-related variables are victimization type, legal stage, and years passed. It is hypothesized that seriously wounded victims and their families will have more unmet needs than bereaved families since seriously wounded victims might have more pressing issues concerning physical rehabilitation and daily recovery tasks. Victims who are at stages before or during court processes are hypothesized to have more unmet needs since the burden of criminal justice participation might elicit new needs which might not be properly addressed. On the other hand, the number of years passed is not hypothesized to be an influencing factor given that all respondents are victims of serious violence or their families and whose trauma could be lifelong.

Victimization impact variables are from answers to reduced household income, relocation, occupational impact, family life impact, and most affected. It is conceivable that the more post-crime impacts perceived, the more likely the victims have a greater demand for services that have not been received. However, whether the victims are satisfied with the service delivery is not presumed to be related to post-crime impacts. That is, the impacts are hypothesized to be associated with unmet needs, but not levels of satisfaction.

Service utilization variables are the number of utilized service agencies and the usage of legal service, financial service, living service, psychological service, and medical service. It is hypothesized that the more services used, the less likely victims will report unmet needs. Furthermore, the more services used is supposed to be positively related to satisfaction levels since greater utilization of services means more information on and access to services. Service satisfaction increases as a result, as previous studies suggest.

### Statistics analysis

The relationship between dependent and independent variables are modeled in the following way:

$$\begin{array}{l} y=\mathrm{gender}+\mathrm{education}+\mathrm{emplyment}+\mathrm{cohabitants}+\mathrm{no}.\;\mathrm{of}\;\mathrm{dependents}+\mathrm{victim}\;\mathrm{type}\\ \;+\mathrm{legal}\;\mathrm{stage}+\mathrm{no}.\;\mathrm{of}\;\mathrm{years}\;\mathrm{passed}+\mathrm{reduced}\;\mathrm{household}\;\mathrm{income}\\ \;+\mathrm{relocation}+\mathrm{occupational}\;\mathrm{impact}+\mathrm{family}\;\mathrm{life}\;\mathrm{impact}+\mathrm{most}\;\mathrm{affected}\\ \;+\mathrm{no}.\;\mathrm{of}\;\mathrm{service}\;\mathrm{agencies}+\mathrm{legal}\;\mathrm{assistance}+\mathrm{financial}\;\mathrm{assistance}\\ \;+\mathrm{living}\;\mathrm{assistance}+\mathrm{psychological}\;\mathrm{assistance}+\mathrm{medical}\;\mathrm{assistance} \end{array}$$

where the dependent variable *y* is log(*p*/(1–*p*)), i.e. logarithm of the odds of unmet needs in the case of unmet needs regression modeling, and *y* is the satisfaction levels in the case of service satisfaction regression modeling. The independent variables on the right-hand side of the equation are, respectively, the five demographic variables, three incident-related variables, five victimization impact variables, and six service usage variables introduced above. Among the 19 independent variables, *education*, *no*. *of dependents*, *no*. *of years passed*, and *no*. *of service agencies* are numerical. The rest are categorical. Multicollinearity of the 19 independent variable is examined by the method of variance inflation factor (VIF) [27]. All of the individual VIF values are below 2.0, suggesting adequate independence of the variables [28]. SAS (version 9.4; SAS Institute, Inc., Cary, NC, USA) is used for the analysis. The results of the descriptive and regression analysis are shown in Tables 1 to 4 in the following section.

**Table 1:** Characteristics of the population, *N* = 312.

Variables		N	%
**Gender**			
	**Female**	**168**	**53.8**
**Education**			
	**Elementary or below**	**56**	**17.9**
	**Junior high**	**87**	**27.9**
	**Senior High**	**120**	**38.5**
	**College**	**44**	**14.1**
	**Graduate or above**	**5**	**1.6**
**Employment**			
	**Not employed**	**180**	**57.7**
**Cohabitants**			
	**spouse/partner**	**121**	**38.8**
	**children/parent**	**141**	**45.2**
	**other relatives/friends**	**21**	**6.7**
	**Live alone**	**29**	**9.3**
**Dependents**			
	**None**	**159**	**51.0**
	**One**	**61**	**19.6**
	**Two**	**66**	**21.2**
	**Three**	**20**	**6.4**
	**More than four**	**4**	**1.3**
	**No answer**	**2**	**0.6**

**Table 2:** Victimization experience and impact, *N* = 312.

Variables		N	%
**Type**			
	**Victims of violence**	**73**	**23.4**
	**Families of victims of violence**	**48**	**15.4**
	**Families of homicide victims**	**191**	**61.2**
**Legal stage**			
	**Before verdict**	**58**	**18.6**
	**After verdict**	**235**	**75.3**
	**others**	**19**	**6.1**
**Reduced household income**			
	**Yes**	**102**	**32.7**
	**No**	**142**	**45.5**
	**Don't know/No answer**	**68**	**21.8**
**Relocation**			
	**Never**	**233**	**74.7**
	**Once**	**65**	**20.8**
	**Twice**	**11**	**3.5**
	**Three times**	**3**	**0.9**
**Occupational impact**			
	**Yes**	**161**	**51.6**
	**No**	**51**	**16.3**
	**Don't know/No answer**	**100**	**32.1**
**Family life impact**			
	**Yes**	**275**	**88.1**
	**No**	**30**	**9.6**
	**Don't know/No answer**	**7**	**2.2**
**Most affected**			
	**Interrupted work**	**27**	**8.7**
	**Disrupted social relationship**	**10**	**3.2**
	**Reduced income**	**118**	**37.8**
	**Disrupted familial relationship**	**8**	**2.6**
	**Changed familial atmosphere**	**62**	**19.9**
	**Impaired physiology**	**13**	**4.2**
	**Lawsuits**	**15**	**4.8**
	**Others**	**31**	**1.9**
	**None**	**22**	**7.1**
	**No answer**	**6**	**9.9**

**Table 3:** Use of services, *N* = 263 (service usage)*, 312 (unmet needs).

Variables	*N*	%
**Legal service usage**	**199**	**75.7**
**Financial service usage**	**169**	**64.3**
**Living service usage**	**42**	**16.0**
**Psychological service usage**	**188**	**71.5**
**Medical service usage**	**15**	**5.7**
**Unmet needs**	**207**	**66.3**

*49 individuals did not respond about service usage.

**Table 4:** Regression models.

	Unmet needs (logistic regression)			Services Satisfaction (linear regression)		
	OR	95% CI	p value	coef	95% CI	p value
**Female**	1.72	[0.541~5.46]	0.359	-0.263	[-3.07~2.55]	0.855
**Education**	0.98	[0.611 ~1.57]	0.932	0.792	[-0.418~2.00]	0.203
**Employment**	0.464	[0.120 ~1.80]	0.266	-1.46	[-4.91~1.99]	0.41
**Cohabitant (alone)**						
Spouse	0.741	[0.120~4.56]	0.746	-5.72	[-10.9~-0.557]	0.0326*
Parent/children	0.909	[0.151~5.47]	0.917	-4.52	[-9.59~0.552]	0.0842^.^
Relatives/friends	22700000	[0~Inf]	0.993	-6.18	[-13.7~1.37]	0.112
**Dependents**	1.17	[0.727~1.89]	0.513	0.296	[-0.977~1.57]	0.65
**Victims of Violence**						
Families of the victims of Violence	0.295	[0.0588~1.48]	0.139	-3.34	[-7.47~0.796]	0.117
Homicide victim families	0.315	[0.0664~1.50]	0.147	-5.18	[-9.13~-1.24]	0.0116*
**Before verdict**						
After verdict	0.295	[0.0809~1.08]	0.0646^.^	3.78	[0.855~6.71]	0.0131*
Others	0.263	[0.0379~1.82]	0.176	8.11	[3.36~12.9]	0.0012**
**Years passed**	0.96	[0.823~1.12]	0.605	-0.366	[-0.753~0.0209]	0.067^.^
**Reduced income**	1.36	[0.494~3.73]	0.554	-3.16	[-5.81~-0.508]	0.0218*
**Relocate**	1.12	[0.459~2.73]	0.805	2.35	[0.165~4.53]	0.0378*
**Occupation impact**	2.19	[0.691~6.97]	0.182	-4.91	[-8.04~-1.78]	0.00283**
**Family life impact**	0.709	[0.116~4.33]	0.709	-0.209	[-4.62~4.21]	0.926
**Most affected (none)**						
Work	0.436	[0.0333~5.69]	0.526	0.454	[-5.77~6.68]	0.887
Social relationship	6280000	[0~Inf]	0.995	3.43	[-10.0~16.9]	0.619
Income	0.501	[0.0559~4.49]	0.537	4.49	[-0.865~9.85]	0.104
Familial relationships	0.632	[0.0214~18.7]	0.791	-0.555	[-8.52~7.40]	0.892
Familial atmosphere	0.3	[0.0344~2.62]	0.276	1.58	[-3.71~6.87]	0.56
Physiology	0.0648	[0.00247~1.70]	0.101	-6.86	[-15.8~2.10]	0.137
Lawsuits	0.71	[0.0504~10.0]	0.8	5.79	[-0.726~12.3]	0.0851^.^
Others	2.37E-09	[0~Inf]	0.996	2.67	[-10.7~16.0]	0.696
**Service agencies**	0.844	[0.447~1.59]	0.601	0.283	[-1.44~2.01]	0.749
**Legal service**	0.81	[0.268~2.45]	0.709	4.99	[2.29~7.69]	0.00048***
**Financial service**	0.997	[0.370~2.68]	0.995	4.25	[1.73~6.78]	0.00138**
**Living service**	0.652	[0.183~2.32]	0.508	0.329	[-2.92~3.58]	0.843
**Psychological service**	1.04	[0.360~3.03]	0.936	3.9	[1.30~6.49]	0.00411**
**Medical service**	0.311	[0.0411~2.35]	0.257	-8.29	[-14.0~-2.55]	0.00575**

Note

(.)p<0.1

(*)p<0.05

(**)p<0.01

(***)p<0.001,

AIC:200.82, 178 observations deleted due to missingness (Logistic Regression); Multiple R-squared:0.5774, Adjusted R-squared:0.435, 192 observations deleted due to missingness (Linear Regression)

## Results and discussion

### Results

#### Characteristics of the population

Victims of violent crime in this survey, both direct and indirect, are more likely to be female (53.8%) with senior high school education (38.5%), unemployed (57.7%), living with children or a parent (45.2%), and without any dependents under 18 years of age (51.0%) (see Table 1).

#### Victimization experience

The time since the violent incident ranged from less than one year to 17 years, with an average of 4.7 years (SD = 2.99). Surviving families of homicide are the majority (*N* = 191, 61.2%), followed by violent assault victims (*N* = 73, 23.4%) and violence victim families (*N* = 48, 15.4%). More than three fourths of the victims have received a verdict (see Table 2).

As to consequences of violent crime, family life impact was chosen by most victims (88.1%), followed by occupational impact (51.6%) and reduced household income (32.7%). Around one fourth moved more than once after the violent incident. When asked which one is the most devastating impact, however, more than one in three had chosen income reduction (37.8%), followed by a changed familial atmosphere (19.9%) (see Table 2).

259 victims (83%) reported having received at least one type of service, another 49 (15.7%) refused to answer, and only four (1.3%) reported never having received any of the five types of services since the victimization event. The number of agencies from which they received services ranges from zero to five. On average, each interviewee received services from 1.17 victim support agencies (SD = 0.82).

The services victims received, from most to least used, are: legal, psychological, financial, living, and medical service. Excluding the 49 interviewees who refused to answer whether they had used these services, the percentages of those using the services are 75.7% legal, 71.5% psychological, 64.3% financial, 16% living, and 5.7% medical (see Table 3).

Although more than 80% of the interviewees had received at least one type of service, as many as 66.3% reported wishing to have had more services. The count of unmet needs could range from zero to fifteen. Among the 207 interviewees reporting having needs unmet, the average count of unmet needs is 2.3. Lastly, the satisfaction levels of services range from 7 to 35. The mean satisfactory level is 23.6 with a standard deviation of 8.22.

#### Unmet needs and service satisfaction

Two regression models were created to help improve our understanding of victims’ unmet needs and service satisfaction. In the unmet needs logistic regression model, only the legal stage factor deserves attention since its *p* value is less than 0.1. As hypothesized, victims or families who are at the stage after court verdict, compared with those before, are slightly less likely to express unmet needs. Neither the personal demographic characteristics nor use of services is found to predict unmet needs (see Table 4).

Many factors are found to associate with service satisfaction in the linear regression model. It is not surprising to find that neither gender, education, and employment are associated with satisfactory level, which is consistent with the assumption. Compared with lone victims/families, victims/families who lived with their spouses/partners or parents/children were less satisfied with the services.

As to the peri-crime factors, homicide families reported less satisfaction with the services than violently wounded victims. If the interviewees had received the verdict or were at other stages of court process, they were significantly more likely to report higher satisfaction than those who had not received the verdict. Both interviewees with reduced household income and occupational impact, compared with no such impacts, were negatively associated with service satisfaction. It is surprising to find that the number of moving after the event, on the other hand, is positively associated with satisfactory level.

It is worth noting that if the interviewee considered the most affected life aspect to be “lawsuit”, he or she was more satisfied with the services, compared with those who did not choose any most affected impact. This might be because legal aid is among the most easily accessible services, relative to financial aid or psychological service. The linear regression model of service satisfaction reveals that receiving legal service is highly predictive to higher satisfaction. Victims who received financial and psychological support are also more likely to report higher satisfaction. Although medical service is also a predictive factor, it is negatively correlated with the level of satisfaction, suggesting that the few victims who needed and received medical service, compared with those who did not, are less satisfied with victim services overall.

## Discussion

### Characteristics of service users

Firstly, descriptive analysis shows that the victim service users in Taiwan are similar to those in Pennsylvania [4] in terms of gender, education, and occupation. Females who did not live with their spouse or partner, with middle level education and without paid full-time employment, might be more likely to become help recipients. In Taiwan, homicide surviving families were more likely to take up and stay in official victim support services. This might be understood in the sense that community-based support groups or private non-profit organizations for the bereaved are rare in Taiwan.

Secondly, the statistics of victimization impacts suggests that nearly nine out of ten victims/families reported suffering from disrupted family life and one in five perceived that changed familial atmosphere as the most disturbing aftermath of the violent incident. This is probably because 61.2% of the interviewees were families of homicide who had permanently lost their loved ones. However, existing literature seldom recognizes that victimization creates burdens for the household and affects every member of the family. Reconstruction of the family life ought to be emphasized at least as much as recovery of individual psychology.

Third, it is worth noting that less than half of the interviewees had paid jobs at the time of the survey, suggesting that some victims might lose their jobs post-crime. Voluntarily or forcedly leaving employment also means disconnecting social networks and withdrawing from social participation, both of which might be negative for rehabilitation. In addition to the occupational change, which was answered “yes” by more than half of the interviewees, “reduced household income” was chosen by most of the interviewees as the most affected in their lives in the survey. The negative occupational and income impacts found in the current study altogether represent financial loss. Hobfoll and Lilly propose the “conservation of resources theory” which stresses the strong relationship between resource loss and stress reaction [29]. According to the theory, people who lose their resources will undergo further resource loss and psychological stress since future resource gain is built upon existing resources. A survey to 810 natural disaster victims found that financial loss to be a determinant of post-disaster PTSD since the loss is a tangible stressor and can multiply to become chronic [30]. Conservation of resources theory can explain why job loss or income loss will create chronic stress following the acute stressful victimization event. In sum, nearly one in three service users fell in a disadvantaged and vulnerable psychoeconomic situation, requiring concern from social welfare agencies. This is consistent with the findings from the New York Victim Service Agency study, which concluded that those who suffered from greater life stress were more likely to be service users [31].

### Unmet needs

Unlike previous studies which often found that service users were only a small group in the victim population [17], this survey reports services take-up rate as high as 83%. Among the service users, higher rates of reporting receiving legal, psychological and financial help were found. This could be explained by the nature of the current population consisting of only serious violence victims and homicide families, and both are categorized as the “neediest victims” [31]. It is of note that victims/families appreciated very little about living service and medical service from the victim support agencies, perhaps because this kind of help can be provided by existing social support groups/establishments, such as relatives, friends, neighbors, and hospitals. The findings confirm that legal, psychological, and financial assistance remain the three predominant services in current victim support in Taiwan [32].

The findings also agree with previous research which suggests that service provision itself would elicit more needs [33]. The percentage of victims/families who report unmet needs in the current study is much higher (66.3%) than that of the general victim population (13% to 24%) found by Davis et al. [16], reconfirming that victims of violence should be a priority for victim support. The results also reflect the fact that victims’ expectations escalate once they actually benefit from the services, together with lingering vulnerability. Moreover, the current logistic regression model demonstrates that the victims/families who had already received the verdict would be less likely to report unmet needs, consistent with the above descriptive finding that the legal service is the most highly sought service among the five services.

### Service satisfaction

Many demographic, crime-related, post-crime impact variables are found to covary with the satisfaction. The findings show that service users who were direct violence victims, lived alone, received the verdict, and relocated, had a relatively greater satisfaction than those who were homicidally bereaved, lived with their spouse, and suffered from income reduction or occupational impact. This suggests that the current uniform and standard service provision might not be equally accessible or equally helpful from the myriad points of view of heterogeneous users. The homicidally bereaved might find their presence in the criminal justice system to be less than the accused, thus causing dissatisfaction. Married users found to receive less post-flood service than their single counterparts in an empirical discussion on older victims of natural disaster [34]. Indeed, Logan, Stevenson, Evans, & Leukefeld [18] concluded that one of the sources of women’ barriers to health and criminal justice services was actually their spouse, who might directly refuse their access to services or indirectly keep women from getting resources by the use of children or home obligations as an excuse. Similarly, it is plausible that income reduction and employment disruption might become barriers to receiving services since victims might choose to earn more money to fill the financial loss or preserve existing social support, rather than pursue unforeseeable and sometimes disappointing new resources. Thus, the lower dissatisfaction reported by victims who were homicidally bereaved, lived with the spouse, and suffered from income reduction or occupational impact, might be better explained by the failure of recognizing the burden of victims who are in need of investing in other resources after their unfortunate loss [26].

The current findings also corroborate previous literature stating that victims’ service usage is of central importance for higher satisfaction [16]. Victims are generally more satisfied with the service when they receive the service. The current study confirms previous studies that address the significance of “outreach” as the key element of effective service delivery [19]. The current findings further mirror Dunn’s conclusion that available services dictate victims’ needs [19].

## Conclusions

This study adds to the scant empirical studies of violence victims and their use of victim services with an Asian flavor. It is of significance since Taiwan is among the first few societies in East Asia that have a Crime Victim Protection (Support) Act (1998) to provide state compensation and support for serious violence victims [35]. This study also constitutes an attempt to evaluate the 1998 legislation by providing a picture of violence victims’ situations from their feedbacks.

The findings shed light on future works toward a better support for the victims of violent crime. In particular, the fact that the majority (83%) of the violence victims in this survey received some kind of services yet still a majority (66.3%) of them reported insufficient services prompts, instead of a “failed” victim support, a rethinking of the current services. My finding suggests that some victims did not receive services not because they did not need services, but because their individual circumstances hindered their access to the needed services. As Dunn states in his literature review [19],“Services to victims of crime should not be based solely on the concept of need, but designed to uphold victims’ rights as well”.

It is thus suggested that victim support policy should go beyond “doing good” toward “doing justice” by relieving the victim’s burden in obtaining services, complemented by viewing service handling as “rights” instead of “welfare” for the victim.

In countries with mature victim support, a plethora of private non-profit organizations and victim-initiated support groups play a significantly major role. My findings also stress the importance of collaborating and coordinating the roles of victim support. Victim support should not be approached solely by meeting the potentially unlimited and ever-lasting needs of victims. Instead, victim support should play a coordinating role in delivering services through “case management”, which functions to remove or lessen obstacles and enables victims of violence to link to a variety of resources according to their own wishes. A better victim support will also involve training practitioners in social work, medical services, and legal services, including victim sensitivity and empathy, in order to prevent secondary victimization. By empowering victims during the process of service provision and with an equal relationship between service provider and recipient, victim support helps the victim to recover their own strength and resilience.

### Research limitations

The study is limited in several respects. First, the data is retrospective and the memories of the victims and families interviewed might fade with the passing of time. Secondly, understanding the use and evaluation of the victim services could be improved by multi-source data collection, including qualitative data with in-depth interviews through a longitudinal survey. Thirdly, the regression model of unmet needs was not as powerful as the satisfaction model, because the distribution of answers to unmet needs displayed a right-skewed distribution bounded at zero rather than a broad, symmetric distribution of the answers to satisfaction. And lastly, the samples include unidentified victims whose cases remained unsolved at the time of interview. Given this limitation of the data, different needs of victims with unsolved cases might not be well addressed, which needs future research.

### Policy implications

The subjects of this study are violent crime victims or their families, whose lives were affected in many respects, including “psychological, academic, social, vocational, and familial adjustment” [7]. This study emphasizes the importance of direct feedback from service recipients in victim support policy-making. It calls for more thought on the role of current victim support as perceived by victims of non-traffic violence as insufficient and far from satisfactory. Several policy implications are proposed within the Taiwanese context, providing reference to other areas in further improvement of victim support practices.

Firstly, the population in this study consists of those victims of non-traffic violence whose incidents dated back 17 years, indicating the longevity of the violence victimization process. Therefore, victim support organizations should be aware of the potentially lingering effects of violence victimization, preventing premature service disengagement from the victims. In addition, more studies, especially longitudinal ones on the victims of extreme violence, are needed for improved victim support.

Secondly, the findings echo what Vincent et. al. suggested a comprehensive model for supporting homicide and violent survivors which integrates specialized relationship counseling, individual counseling, or group counseling following immediate crisis intervention services [36]. Empirical studies have found that children in families that suffered from violent crime are at higher risk of PTSD, depressive episodes, and drug abuse/dependence [37]. A qualitative analysis of 38 families which suffered from public environment violence confirmed deteriorated family function effects such as communication problems and family conflict [38]. Although a disrupted familial relationship is easily neglected in the early days of violence victimization, the negative effect will grow to become substantial. Assistance which focuses on rebuilding the family and social relationships might actually also improve a victim’s mental and physical health, reducing additional household burdens. It is also suggested that psychological help, such as counseling, following practical assistance, and aiming to: (a) relieve the pressing household burdens; and (b), build trust between practitioners and victims, can be much more effective [21].

Thirdly, victim support in Taiwan has achieved a great deal in terms of reaching out to the neediest victims of violence. To differentiate groups of victims of violence and to hear their voices in the decision-making process might be the next step to improve service delivery. The services could be further improved by early assessment of the victims and their families in order to layout a long-term rehabilitation plan. Special plans should be tailored for the victims whose offenders have never been found or were not prosecuted, since these victims are often prematurely discharged from the victim services once their involvement in the criminal justice system ends. The study draws attention to the importance of dialogues between victims of violence and victim support practitioners about service users’ outlook on future life and their own active roles in it.

In 2009, the Crime Victim Protection Act of Taiwan was modified, expanding its coverage to include victims of sexual assault, domestic violence, and human trafficking. Victims who are minors or non-citizen spouses/workers have also been included for protection. These victims are more likely to encounter service barriers and be kept away from the service provision. More studies focusing on these potentially disadvantaged victims are needed.

## Supporting information

S1 Text Survey questionnaire (in Chinese and English translation). (XLSX)

S1 Data File of 312 samples. (CSV)
